# Characterization of Thermotolerant Chitinases Encoded by a *Brevibacillus laterosporus* Strain Isolated from a Suburban Wetland

**DOI:** 10.3390/genes6041268

**Published:** 2015-12-04

**Authors:** Pulin Liu, Deyong Cheng, Lihong Miao

**Affiliations:** College of Biological and Pharmaceutical Engineering, Wuhan Polytechnic University, Wuhan 430023, China; E-Mails: liunan3585@163.com (P.L.); SG201302002@163.com (D.C.)

**Keywords:** *Brevibacillus laterosporus*, chitinase, kinetic analysis, enzymatic properties

## Abstract

To isolate and characterize chitinases that can be applied with practical advantages, 57 isolates of chitin-degrading bacteria were isolated from the soil of a suburban wetland. 16S rRNA gene analysis revealed that the majority of these strains belonged to two genera, *Paenibacillus* and *Brevibacillus*. Taking thermostability into account, the chitinases (ChiA and ChiC) of a *B. laterosporus* strain were studied further. Ni-NTA affinity-purified ChiA and ChiC were optimally active at pH 7.0 and 6.0, respectively, and showed high temperature stability up to 55 °C. Kinetic analysis revealed that ChiC has a lower affinity and stronger catalytic activity toward colloidal chitin than ChiA. With their stability in a broad temperature range, ChiA and ChiC can be utilized for the industrial bioconversion of chitin wastes into biologically active products.

## 1. Introduction

Wetlands are one of the most productive natural ecosystems on Earth—they perform various ecological functions, including nutrient cycling, carbon storage, flood reduction, and provision of habitats for wildlife [1]. Microorganisms are vital components of wetlands, responsible for many key processes. The high diversity and abundance of bacterial species in wetlands make this environment an ideal source for the isolation of bacteria with biotechnological applications. 

Chitin, a β-(1,4)-linked polymer of *N*-acetyl-_D_-glucosamine, is the second most abundant carbohydrate polymer in nature and is a major structural polysaccharide of arthropods, plankton, and fungi [2]. Chitinolytic bacteria, ubiquitous in soil, play a key role in the decomposition of chitin in wetland ecosystems [3]. Chitinases secreted by these microorganisms can efficiently convert chitin biomass into fermentable sugars. Compared to chemical conversion, chitinase-mediated conversion is regarded as a green and environmentally friendly technology. Bacterial chitinases generally consist of multiple functional domains, such as the carbohydrate-binding module domain (CBM), the fibronectin type III-like domain, and the catalytic domain [2]. The importance of CBMs in the degradation of colloidal chitin has been demonstrated in some bacterial chitinases [4]. Many bacteria, including *Bacillus circulans* [5], *Streptomyces lividans* [6], *Aeromonas* sp. [7], and *Serratia marcescens* [8,9], produce multiple chitinases from different genes, and the efficient degradation of chitin is assumed to be achieved by the combined action of chitinases [10]. 

In this study, the chitinolytic bacteria from a suburban freshwater wetland were isolated. Among these bacteria, one *Brevibacillus laterosporus* strain containing two chitinase genes demonstrated strong chitinolytic activities at high temperature. To determine the individual roles of ChiA and ChiC from this strain, the two chitinases produced by *Escherichia coli* cells were purified, and their enzymatic properties were analyzed.

## 2. Experimental Section

### 2.1. Sample Collection and Chitinolytic Bacteria Isolation

The wetland located north of Wuhan City (114°18'E, 30°38'N), China, was selected as the sampling site of this study. Ten soil sediment samples were collected from discrete locations at the end of October 2013. The samples were placed in clean and sterile plastic bags, transported in an ice bath to the laboratory within 3 h after sampling, and stored at 4 °C for bacterial colony counting and chitinolytic bacteria isolation. The chitinolytic bacteria were isolated as described by Hoster *et al.* [11].

For bacterial colony counting, 10 g of fresh soil sediment was suspended in 90 mL of sterilized phosphate-buffered saline. This suspension was then serially diluted tenfold. From each dilution, 100 μL of the suspensions were aliquoted and spread on five plates containing the solid medium reported by Yang *et al.* [12] (10 g of peptone, 10 g of yeast extract, 5 g of NaCl, 1 g of K_2_HPO_4_, 1 g of MgSO_4_, and 15 g of agar per liter). The number of culturable bacteria was counted after incubation at 30 °C for 3–7 days. 

### 2.2. Preparation of Colloidal Chitin

Colloidal chitin was prepared from commercial chitin via the method described by Roberts and Selitrennikoff [13]. Twenty grams of chitin powder was slowly added to 350 mL of concentrated HCl and left at 4 °C overnight with vigorous stirring. One liter of 95% ethanol was added to this suspension, and the resulting mixture was centrifuged at 5000 g for 10 min. The pellet was washed with distilled water until the pH reached 6.5, and was then dried by lyophilization.

### 2.3. Fermentation and Chitinase Activity Assay

One hundred milliliters of the fermentation medium modified from Hoster *et al.* [11] (colloidal chitin, 7.0 g·L^−1^; yeast extract, 2.0 g·L^−1^; KH_2_PO_4_, 3.0 g·L^−1^; K_2_HPO_4_, 2.0 g·L^−1^; NH_4_Cl, 1.0 g·L^−1^; MgSO_4_·7H_2_O, 0.2 g·L^−1^; CaCl_2_·2H_2_O, 0.01 g·L^−1^) was transferred to individual 250 mL baffled flasks and autoclaved. The flasks were inoculated with 1 × 10^6^ cells and fermented at 30 °C for 48 h in an orbital shaking incubator. The supernatants were obtained from the culture medium by centrifugation at 13,000 g for 10 min.

Chitinase activity was assayed using colloidal chitin as the substrate. The supernatant or the enzyme solution (2 mL) was added to the substrate solution, which contained 2.5% colloidal chitin in phosphate buffer (20 mmol·L^−1^). The mixture was incubated at different temperatures for 1 h. After centrifugation, the concentration of reducing sugar was measured by the dinitrosalicylic acid method [14]. The reducing sugar levels were determined relative to the *N*-acetyl-β-_D_-glucosamine standards of 10 to 100 μg·mL^−1^. One unit of chitinase activity was defined as the amount of enzyme required to produce 1 μmol of reducing sugar per minute at 30 °C. The measured absorbance values were corrected against blank absorbance readings.

### 2.4. Bacterial Total DNA Extraction and 16S rRNA Gene Sequencing

Sampled bacteria were cultured in liquid broth for 24 h, and cells were then harvested and subjected to genome DNA extraction by using a TIANamp Bacterial DNA Kit (Tiangen Biotech., Beijing, China). The 16S rRNA genes of the isolates were amplified by PCR with the primer set of 27F and 1492R (Table 1). The PCR products were directly cloned into pMD18-T by using a TA cloning kit (Takara, Dalian, China). Subsequently, the inserting fragments were sequenced and analyzed. The sequences were aligned using CLUSTAL X [15], and the alignments were written in a CLUSTAL format file. Using the information from this file, a neighbor-joining tree was constructed by MEGA 4.0 [16] with the substitution model of Kimura-2.

**Table 1:** Primers used in this study.

	Primer Sequence ^a^	Product Length (in bp)
27F	aga gtt tga tcc tgg ctc	—
1492R	ggt tac ctt gtt acg act	
*chiA* cloning	atg aaa aga ttt ttc ntc atg gc	1950
	cta tga ggt tcc tgt aat kgg tg	
*chiC* cloning	atg tat caa cac att cct act g	2574
	tta ctt atc agt aac cgc ata t	
ChiA expression	gcc gag ctc gag aac agg agc aac tcc tc	1875
	ccg ctc gag cta tga ggt tcc tgt aat kgg tg	
ChiC expression	gcc gag ctc tcg ycg atc tgc tca gct g	2460
	ccg ctc gag tta ctt atc agt aac cgc ata ttt ttt g	

^a^ underlined are restriction sites.

### 2.5. Heterologous Expression of Chitinase from B. lateroporus M64

*ChiA* and *chiC* were amplified from genomic DNA at an annealing temperature of 55 °C by using gene-specific primers (Table 1). The expression vector pET28a (Novagen, Madison, Wisconsin, USA) and amplicons were digested by *Sac*I and *Xho*I, purified with gel, and ligated by T4 DNA ligase at 16 °C for 12 h. Recombinant plasmids were designated as pET28-chiA and pET28-chiC. The signal peptide was predicted using SignalP 4.0 [17]. Both genes were cloned without the signal peptide-encoding portion. The recombinant *chiA* and *chiC* were expressed in *E. coli* BL21 (DE3) (Novagen, Madison, WI, USA). The isolated strain M64 has been deposited at the China Center for Type Culture Collection under the accession number AB2015253.

### 2.6. Purification of Chitinases and Their Characterization

*E. coli* BL21 (DE3) strains containing pET28-chiA or pET28-chiC were grown in 100 mL of Luria-Bertani medium supplemented with kanamycin (50 μg·mL^−1^) at 37 °C under shaking conditions (180 rpm). When the absorbance reached OD = 0.4 to 0.6, the cultures were cooled and incubated at 16 °C for 30 min. Isopropyl-β-thiogalactopyranoside was then added to a final concentration of 0.1 mmol·L^−1^, and the cultures were successively shaken at 16 °C for 12 h. The cells were harvested by centrifugation, suspended in 10 mL of 50 mmol·L^−1^ sodium phosphate buffer, and disrupted by sonication. Cell debris was removed by centrifugation at 12,000 g for 10 min. Recombinant ChiA and ChiC proteins were purified from the supernatant by using Ni-NTA chromatograph and desalinated with Sephadex G-25 (Shenggong, Shanghai, China). Protein concentration was determined by Bradford method using a protein quantitative assay kit (Shenergy Biocolor, Shanghai, China) with bovine serum albumin as standard.

Chitinase activities under different pH conditions were determined in various pH buffers [18]. Chitinase activities at different temperatures ranging from 20 °C to 80 °C were also measured. K^+^, Mg^2+^, Fe^2+^, Fe^3+^, Cu^2+^, and Hg^2+^ in chloride salt forms were added to the reaction mixtures to achieve a final concentration of 5 mmol·L^−1^ to evaluate the effects of metal ions on enzyme activities. Relative activities were determined under optimum conditions and compared to metal ion-free experiments. The Michaelis-Menten constant (*K_m_*) and maximal reaction velocity (*V_max_*) were calculated using Origin 6.0 (OriginLab Corporation, Northampton, MA, USA) with nonlinear regression. The turnover number (*k_cat_*) was calculated by the following equation: *k_cat_* = *V_max_*/E, where E represents the enzyme concentration [19].

### 2.7. Nucleotide Sequence Accession Numbers

The 16S rRNA gene sequences of the isolates have been deposited in the GenBank database under the accession numbers KR856187 to KR856200. The accession numbers of *chiA* and *chiC* are KR856201 and KT003293.

## 3. Results

### 3.1. Culturable Chitinolytic Bacteria Isolated from Wetland Samples and Phylogenetic Classification 

**Figure 1:**
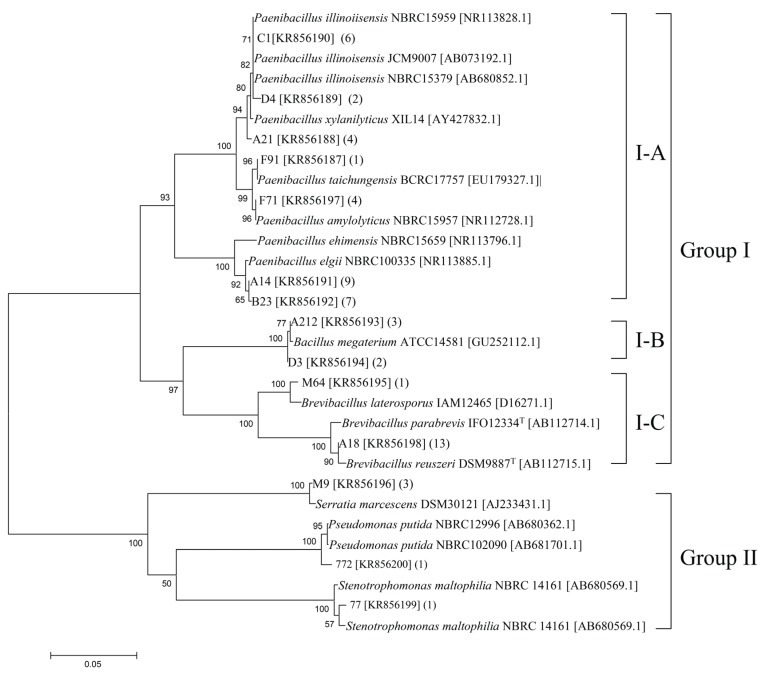
Phylogenetic relationship of the representative chitinolytic bacteria isolated from the wetland based on the aligned sequences of the 16S rRNA gene. Numbers above each node indicate percentage of confidence levels generated from 1000 bootstrap trees. The number of isolates with the same 16S rRNA gene is shown in parentheses.

Numerous colonies were observed on the solid medium after incubation for 3–7 days. The culturable bacterial abundance in different samples varied from 1.2 × 10^8^ to 4.0 × 10^8^ cfu·g^−1^. The identified chitinolytic bacteria represented 1.6% of the total culturable bacteria. Fifty-seven pure culture isolates (14 strains) of chitinolytic bacteria were obtained in this study. On the basis of the 16S rRNA gene sequences, a neighbor-joining tree was constructed to reveal their evolutionary distances and phylogenetic relationships (Figure 1). All of the isolates were roughly classified into two groups. Group I consisted of the majority of the bacteria and accounted for 91% of the total bacterial communities. This cluster could be further divided into three subgroups, denoted as I–A, I–B, and I–C. Phylogenetic analysis revealed that the isolates in this cluster were related to *Paenibacillus* (I–A), *Bacillus* (I–B), and *Brevibacillus* (I–C). Group II accounted for 9% of the bacterial communities. The representative isolates C77, C772, and M9 were phylogenetically related to *Stenotrophomonas maltophilia*, *Pseudomonas putida*, and *S. marcescens*, respectively.

### 3.2. Chitinolytic Activities

The chitinolytic activities of the representative isolates were analyzed through baffled flask fermentation. Most of the strains showed the highest chitinase activity at 36 h and then activity gradually decreased (Supplementary material, Table S1); After 1 h of incubation at 30 °C, the culture supernatant of *Paenibacillus* A21 exhibited the highest chitinase activity (128.9 U·L^−1^), whereas the supernatant of *Paenibacillus* F91 showed the lowest chitinase activity among the isolates (23 U·L^−1^). However, the chitinases secreted by *B. laterosporus* M64 exhibited the highest chitinolytic activity at 60 °C (Supplementary material, Table S2). Therefore, the chitinases produced by *B. laterosporus* M64 and the corresponding genes were selected for further molecular and biochemical characterization.

### 3.3. Cloning and Molecular Analysis of the chiA and chiC Genes from *B. laterosporus* M64

**Figure 2:**
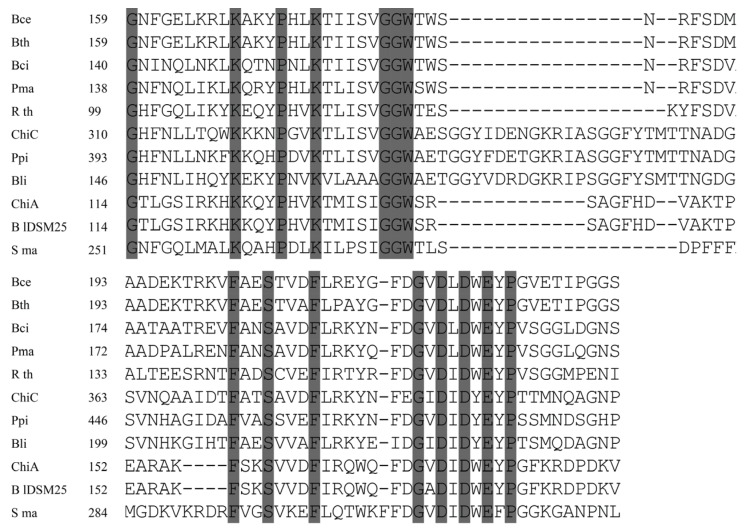
Alignment of the catalytic region from ChiA and ChiC with chitinases from *B. cereus* (Bce, EEK85987.1), *B. thuringiensis* (Bth, EEM24441.1), *B. circulans* (Bci, AAA81528.1), *P. macerans* (Pma, KFM93118.1), *R. thermocellum* (Rth, CAA93150.1), *Pseudoalteromonas piscicidagi* (Ppi, BAB79618.1), *B. licheniformis* (Bli, ACI24006.1), *B. laterosporus* DSM25 (BlDSM25, WP_003336146.1) and *S. marcescens* (Sma, BAA31567.1). Conserved amino acids are shaded. Dashes indicate gaps left to improve alignment. Numbers refer to amino acid residues at the start of the respective lines; all sequences are numbered from the Met-1 peptide.

The chitinase genes of *B. lateroporus* M64 were cloned using isolated genomic DNA as template and two pairs of primers designed from the homologous genes in *B. laterosporus* DSM25 (WP_018670648.1 and WP_018671496.1) and LMG15441 (BRLA_c027050 and BRLA_c008580). Sequence analysis revealed that the ORF of *chiA* consists of 1950 nucleotides. The deduced gene product consists of 649 amino acids with a predicted molecular mass of 72.2 kDa. SignalP 4.0 predicted the presence of a signal peptide with a cleavage site located between Ala^25^ and Glu^26^. ChiA exhibited the typical domain structure of bacterial family 18 chitinases: a triosephosphate isomerase barrel fold with a conserved DxDxE motif [8]. The catalytic region (Gly-114 through Val-189) was found to be 99% identical to that of *B. laterosporus* DSM25. Further analysis showed certain similarities to the catalytic region of other bacterial chitinases, such as those of *Ruminiclostridium thermocellum* (61%) and *B. circulans* (51%). Moreover, the amino acid residues Asp^174^, Asp^176^, and Glu^178^ of ChiA may correspond to Asp^200^, Asp^202^, and Glu^204^ of *B. circulans* ChiA [20], whose role in the catalytic process was previously demonstrated (Figure 2).

**Figure 3:**
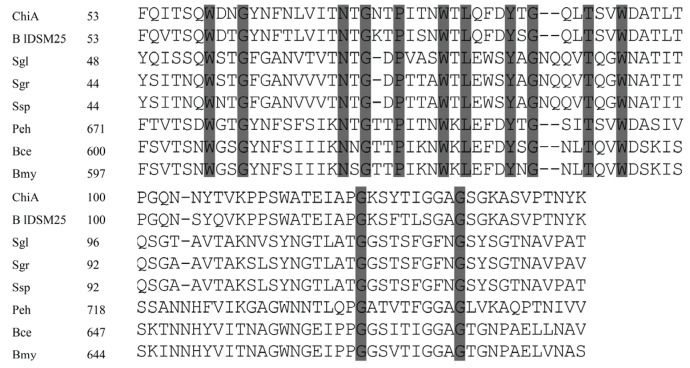
Alignment of CBM in ChiA with the CBMs of the chitinase from *B. laterosporus* DSM25 (BlDSM25, WP_003336146.1), *P. ehimensis* (Peh, BAC76694.1), *B. cereus* (Bce, EEK85987.1), *B. mycoides* (Bmy, EEM01294.1), and the cellulose binding domain of the cellobiosidase from *S. globisporus* (Sgl, WP_030693025.1), *S. griseus* (Sgr, WP_030754441.1), and *Streptomyces* sp. (Ssp, WP_043227939.1). Conserved amino acids are shaded. Dashes indicate gaps left to improve alignment. Numbers refer to amino acid residues at the start of the respective lines; all sequences are numbered from the Met-1 peptide.

At the C-terminus of ChiA, CBM2 displays 52% sequence identity to the sequence of Chi80 (BAC76694.1) from *Paenibacillus ehimensis*, and 51% to ChiC (EEK85987.1) of *Bacillus cereus*. Moreover, the CBM2 of ChiA also shows similarity to the cellobiosidases from *Streptomyces griseus* (accession no. WP_030754441.1; 44% sequence identity) and *S. globisporus* (accession no. WP_030693025.1; 42% sequence identity) (Figure 3). The residues Trp^559^, Trp^580^, and Trp^596^ of ChiA are highly conserved.

The analysis of the deduced amino acid sequence of *chiC* revealed that the modular enzyme is composed of three domains in the following order: a CBM5 domain, a fibronectin-like domain, and a family 18 chitinase domain. The 38 amino acids located at the N-terminus were predicted to be a signal peptide for protein export. The sequence F^152^EGIDIDYE^160^ shows high similarity to the pattern from the PROSITE database for the active-site consensus sequence: [LIVMFY]-[DN]-G-[LIVMF]-[DN]-[LIVMF]-[DN]-x-E, where E is the active-site residue [21]. A fibronectin type III domain (FnIIID), described in many chitinase studies, was identified in amino acids P^103^ and T^186^. FnIIID likely functions as a bacterial carbohydrase spacer to enable the degradation of insoluble substrates [22]. A CBM5 with a conserved sequence AKWWT was located between A^44^ and D^84^. This CBM5 exhibits 58% and 56% sequence identity with the CBMs identified in *S. griseus* (BAA23739.1) and *Nocardiopsis prasina* (BAC45252.1), respectively.

### 3.4. Purification of ChiA and ChiC

**Figure 4:**
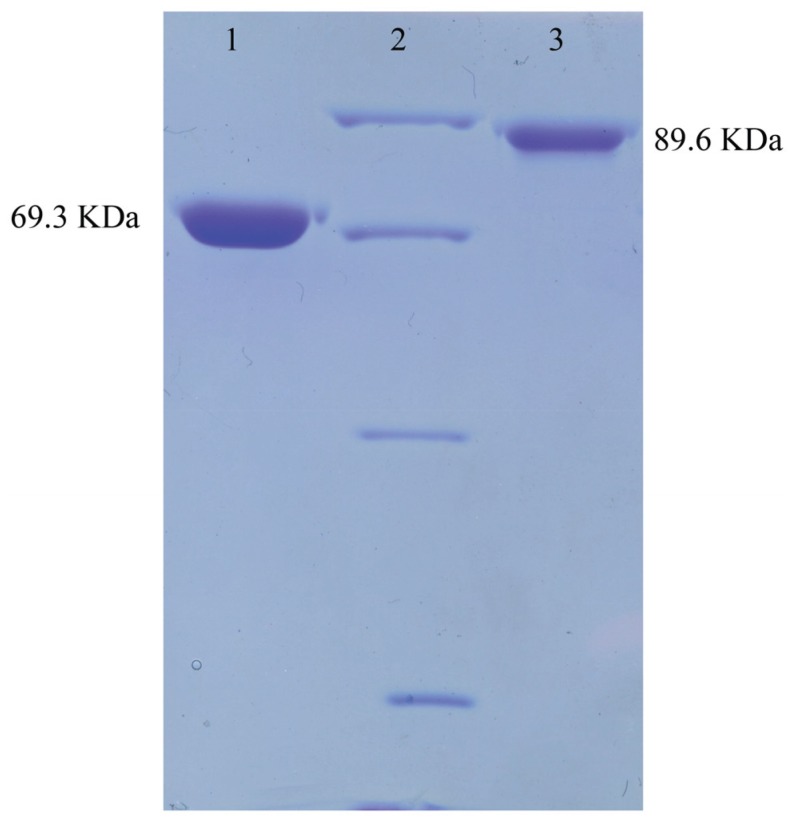
The electrophoresis of ChiA and ChiC. Purified ChiA and ChiC were loaded on 10% SDS-PAGE followed by Coomassie staining. Lane 1, Purified ChiA; lane 2, Middle ranged protein marker (Shenggong, Shanghai, China; 97.4 KDa, 66.2 KDa, 42.7 KDa, 31.0 KDa); lane 3, purified ChiC.

The DNA fragments corresponding to the whole ORF minus the N-terminal signal peptide were amplified, inserted into the expression vector pET28a, and expressed in *E. coli* BL21 (DE3) to analyze the functions of ChiA and ChiC. ChiA and ChiC were successfully expressed in soluble forms and the purified enzymes without the His-tag were obtained after thrombin treatment (Figure 4). The specific activities of ChiA and ChiC were estimated as 22.30 and 63.41 μmol·min^−1^·mg^−1^ by using 2.5% colloidal chitin as substrate.

### 3.5. Effects of Temperature and pH on ChiA and ChiC Activities

**Figure 5:**
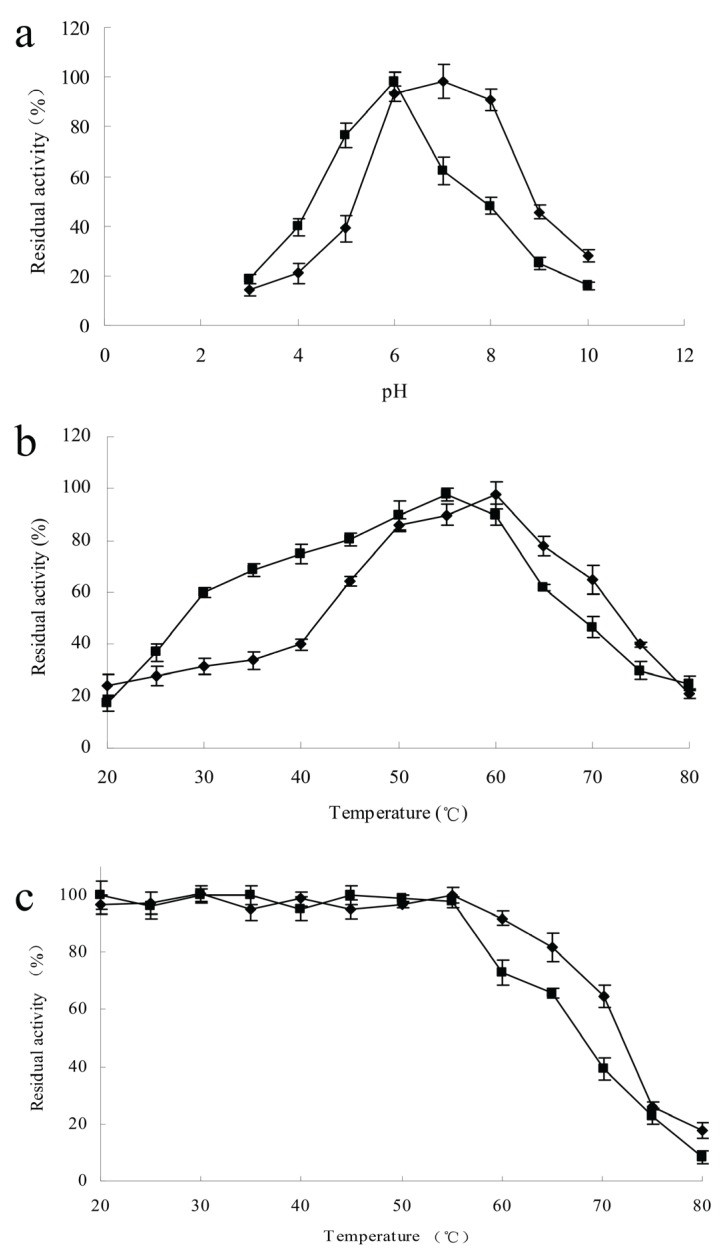
Characterization of ChiA (◆) and ChiC (■) using colloidal chitin as substrate. Each point represents the average of three measurements. (**a**) pH activity profile; (**b**) Temperature activity profile; (**c**) Thermostability profile—The enzymes were incubated for 2 h at the indicated temperatures, after which residual activity was determined and the measurements from incubation at optimum conditions were used as the 100% value.

The effects of pH and temperature on chitinase activities were measured at pH 3.0–10.0 and at 20–80 °C. The highest activities of ChiA and ChiC were recorded at pH 7.0 and 6.0, respectively. The remaining chitinase activity of ChiA was 28% of the maximal observed activity at pH 10.0; in contrast, the remaining chitinase activity of ChiC was 16% (Figure 5a). A hydrolyzing reaction was performed at different temperatures under optimal pH conditions to determine the optimal temperature. The activities of the two chitinases were diminished at temperatures over 60 °C. The optimal temperature for ChiA was slightly higher than that for ChiC. ChiC retained a high percentage of activity at lower temperatures; approximately 59% of the maximum activity remained at 30 °C (Figure 5b). The thermal stability of the enzymatic proteins was investigated in the temperature range of 20–80 °C. After the enzymes were incubated at 55 °C for 2 h, no decline in their activity was observed. The preincubation of ChiA at 60 °C decreased the enzyme activity by 7%; ChiC remained stable only up to 55 °C, and 27% of its activity was lost at 60 °C (Figure 5c).

### 3.6. Effects of Metal Ions on the Activities of Purified Enzymes

Metal ions play a significant role in biological catalysis by forming complexes with the enzymes and maintaining or disrupting the three-dimensional structure and conformation [23]. The influence of metal ions on the activities of chitinases obtained from *B. laterosporus* M64 is presented in Table 2. The activity of ChiA was enhanced by 13% in the presence of Ca^2+^, but was moderately to strongly inhibited by Fe^3+^, Cu^2+^, and Hg^2+^. The presence of Mg^2+^, K^+^, and Fe^2+^ did not significantly affect ChiA activity. In contrast to the activity of ChiA, the activity of ChiC was enhanced 1.15 and 1.09-fold by Ca^2+^ and Fe^2+^, respectively. The heavy metal ions tested inhibited the activity of ChiC to different degrees: Hg^2+^ by 86% and Cu^2+^ by 34%. The ChiC activity remained almost unchanged in the presence or absence of K^+^, Mg^2+^, and Fe^3+^. Cu^2+^ ions can catalyze the auto-oxidation of cysteines to form intramolecular disulphide bridges or sulfenic acid; Hg^2+^ reacts with the –SH groups found in cysteine residues in the protein chain and disrupts the tertiary structure, which strongly inhibits the activities of many enzymes [23].

**Table 2:** Effects of metal ions on chitinase activity.

Metal Ion	Percent Relative Activity (in Percentage)	
	ChiA	ChiC
Mg^2+^	98.83 ± 1.7	101.17 ± 1.8
K^+^	103.75 ± 2.1	97.82 ± 1.1
Ca^2+^	113.85 ± 0.9	115.90 ± 1.4
Fe^2+^	105.38 ± 2.6	109.82 ± 1.9
Fe^3+^	93.13 ± 1.1	96.65 ± 3.1
Cu^2+^	81.86 ± 3.8	66.79 ± 1.8
Hg^2+^	21.50 ± 3.0	13.34 ± 2.5

### 3.7. Kinetic Parameters of ChiA and ChiC

**Figure 6:**
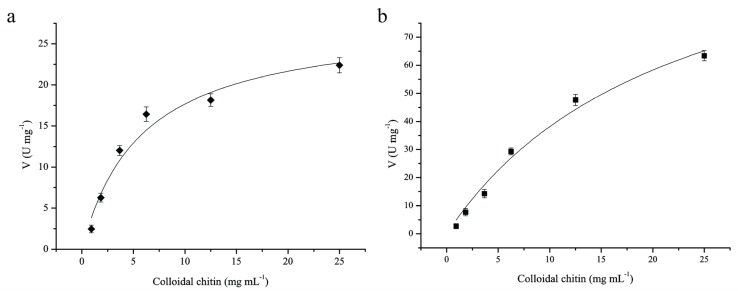
Michaelis-Menten plots of purified chitinases toward colloidal chitin. (**a**), Michaelis-Menten plot of ChiA; (**b**), Michaelis-Menten plot of ChiC; Kinetic parameters were calculated from the initial rate activities of the purified enzymes using colloidal chitin at concentrations ranging from 0.1 to 25 mg·mL^−1^ at optimum conditions.

The kinetic values of ChiA and ChiC were determined with colloidal chitin as substrate. The kinetic parameters were calculated by fitting the hyperbolic curves to the Michaelis–Menten equation using Origin 6.0 (Figure 6). The curve-fitting revealed that the *K_m_* of ChiA and ChiC were estimated as 5.8 and 22.35 μmol·L^−1^, respectively, and the corresponding *k_cat_* were 32.37 and 184.29·S^−1^. Thus, ChiC exhibits a lower affinity to the substrate and a stronger catalytic activity than ChiA.

## 4. Discussion

The characterization of chitinolytic bacteria has received considerable attention because of their important roles in carbon and nitrogen cycling in nature. The detection of chitin-degrading bacteria from natural sources, such as soil and sediment, is also useful to improve the activity of bacterial antibiotics [24,25,26]. Chitinase gene expression in microorganisms has been reported to be controlled by a repressor/inducer system in which chitin or its degradation products act as inducers; repression of chitinase synthesis by glucose is observed in almost all chitinolytic microorganisms [27]. In this study, 57 chitinolytic bacterial isolates were isolated from wetland soil by using colloidal chitin as substrate and indicator. The low and basal levels of chitinase may be sufficient to initiate chitin degradation and to release soluble oligomers, which in turn induced chitinase synthesis [27]. Moreover, soluble inducers cannot be completely removed from colloidal chitin by washing. The analyses of the 16S rRNA gene sequences revealed that the majority of the isolated microorganisms were delineated into *Brevibacillus* and *Paenibacillus*, which accounted for 88% of the total chitinolytic bacteria. This composition is not only different from that found in rhizospheres of agronomic plants, but also greatly differs from the composition encountered in chitin-enriched soils, where *Gammaproteobacteria* appeared to be predominant [28,29].

*B. laterosporus* is a natural inhabitant of water and soil; different *B. laterosporus* strains show a broad-spectrum of antimicrobial activity—many protein toxins (e.g., Vip1Da1, Vip2Ad1, and Vip1Ba1), nonribosomal peptides (e.g., laterosporulin and laterocidin), and antibiotics (e.g., laterosporamine, basiliskamides, and bacithrocins) associated with the observed antagonism have been investigated [30]. However, the enzymatic activities of its chitinases were investigated as a mixture, and their kinetic parameters (*Km* and *k_cat_*) have not been reported [18]. Most bacterial chitinases reported to date belong to family 18 of glycosyl hydrolases (GHs). According to the data from the CAZy database, seven family 18 GHs are present in *B. laterosporus* genomes, including two chitinases, three sporulation-specific glycosylases (YdhD), and two spore germination proteins (YaaH). Family 18 chitinases can be further classified into three subfamilies: A, B, and C [8,31]. On the basis of amino acid sequence similarity, ChiA and ChiC can be classified as members of subfamily A. The main structural difference between chitinase subfamilies A and B is a small (α+β) domain inserted in the catalytic domain, which is present in subfamily A but absent in subfamily B; the inserted domain forms a wall alongside the substrate binding cleft. As a result, the depth of the cleft increases [32]. Considering this structural feature, Li and Greene [33] proposed that chitinases in subfamily A tend to hydrolyze chitin in a processive manner. Enzyme assays with different pH levels revealed that ChiA optimal activity is at pH 7.0, while ChiC was optimally active at pH 6.0. Similar pH optima have been determined in the chitinases from *B. lichenniformis* strain DSM 13 [34] and Chi72 from *B. licheniformis* strain SK-1 [35]. Most chitinases investigated show moderate to high activity as well as stability within the pH range of 4.0–10.0 [36]. A smaller group comprises enzymes that are highly active at pH over 10.0. Such enzymes include the chitinase synthesized by *Streptomyces* sp. with an optimum pH of 12.5, and an alkaline chitinase encoded by *Cellulomonas flavigena* NTOU1 with pH optima at 10.0 [37,38]. Karthik [36] emphasized that the thermostability of most bacterial chitinases is generally up to 50 °C. In this study, the optimum temperature of ChiA and ChiC ranged from 55 °C to 60 °C; ChiA remained stable up to 60 °C. These two enzymes are more stable than the recently described chitinases from *Bacillus halodurans* [21], *Stenotrophomonas maltophilia* [39], and *Streptomyces* sp. [36]. Similar temperature optimum and stability were noted in the case of a 40 kDa chitinase from *Streptomyces* sp. CS495 [38]. The optimum temperature and thermotolerance of ChiA and ChiC are a little lower than those of the chitinase mixture from *B. laterosporus* Lak1210 [18]. After comparing the deduced amino acid sequences of ChiA/ChiC with the short peptide sequences encoded by the homologous genes from *B. laterosporus* Lak1210 [18], we inferred that the amino acid changes in the catalytic domains (K140R and K357R in ChiA; Y417L, P418M, and E419T in ChiC) caused by point mutation or differences in the post-translational modification of proteins between *E. coli* and *B. laterosporus* led to this phenomenon. Many industrial processes operate at high temperatures to increase efficiency and to reduce microbial contamination, which make the enzyme necessary to suit the process requirements. The reasonably high optimum temperatures and their stability facilitate the use of ChiA and ChiC in the degradation of chitin wastes or its bioconversion into biologically active products.

## 5. Conclusions

The community composition of culturable chitinolytic bacteria in the soil of a suburban wetland was resolved through isolate collection. ChiA and ChiC encoded by *B. laterosporus* strain M64 were purified and kinetically analyzed. The purified gene products were active over a wide temperature range, which indicated that ChiA and ChiC are good candidates for chitin waste bioconversion and other biotechnological applications.

## Supplementary Materials

Table S1: Time course of the chitinolytic activity of every strain.

Table S2: The chitinolytic activity of the culture supernatants recorded at 60 °C.
