# Supplementary material for: Characterization of Thermotolerant Chitinases Encoded by a Brevibacillus laterosporus Strain Isolated from a Suburban Wetland
Source: Genes (Basel). 2015 Dec 4;6(4):1268–82. doi: 10.3390/genes6041268 (PMC4690040; doi:10.3390/genes6041268)
Supplement: Supplementary file 1 [file genes-06-01268-s001.pdf]

Supplementary Materials

Table S1. Time course of the chitinolytic activity of every strain.

	**Chitinase Activity of Different Trains (U·L^{-1})**													
Time (h)	C1	D4	A21	F91	F71	A14	B23	A212	D3	M64	A18	M9	C772	C77
12	30.1 ± 0.5	16.2 ± 1.1	45.6 ± 2.3	9.8 ± 1.6	18.8 ± 0.4	9.1 ± 1.1	10.2 ± 2.5	18.4 ± 0.7	19.3 ± 3.5	40.3 ± 1.1	10.4 ± 1.3	40.2 ± 3.4	6.6 ± 0.6	25.2 ± 3.5
24	41.9 ± 2.5	24.4 ± 0.8	68.2 ± 1.2	13.8 ± 1.2	31.3 ± 1.6	11.7 ± 1.5	19.2 ± 1.9	32.2 ± 1.6	37.3 ± 3.0	52.3 ± 2.8	18.4 ± 2.4	58.2 ± 5.1	9.7 ± 0.5	40.2 ± 2.9
36	46.9 ± 2.3	34.6 ± 0.8	128.9 ± 2.5	23.0 ± 0.4	46.0 ± 0.7	41.5 ± 1.4	27.6 ± 1.7	46.1 ± 0.4	52.5 ± 2.1	63.0 ± 1.6	35.9 ± 2.9	58.9 ± 4.7	23.2 ± 1.5	60.9 ± 3.1
48	40.0 ± 1.9	29.3 ± 0.6	100.7 ± 2.7	18.8 ± 2.4	40.2 ± 1.5	35.6 ± 2.8	24.5 ± 2.1	41 ± 3.5	45.5 ± 2.4	54.1 ± 2.7	30.0 ± 1.6	51.6 ± 3.1	10.7 ± 1.3	52.5 ± 2.2

Table S2. The chitinolytic activity of the culture supernatants recorded at 60 °C.

	**Strains**													
	C1	D4	A21	F91	F71	A14	B23	A212	D3	M64	A18	M9	C772	C77
Chitinase activity (U·L^{-1})	50.4 ± 3.7	70.6 ± 2.9	122.5 ± 4.1	16.5 ± 1.4	38.3 ± 2.3	81.4 ± 2.5	48.1 ± 4.0	94.1 ± 3.8	86.0 ± 1.1	134.1 ± 2.4	37.3 ± 3.7	101.6 ± 3.2	11.4 ± 1.6	43.5 ± 1.9
Compared with that in 30 °C (fold)	1.07	2.04	0.95	0.72	0.83	1.96	1.74	2.04	1.64	2.13	1.04	1.73	0.49	0.71

© 2015 by the authors; licensee MDPI, Basel, Switzerland. This article is an open access article distributed under the terms and conditions of the Creative Commons Attribution license (http://creativecommons.org/licenses/by/4.0/).
